# To Resolve or Not To Resolve, that Is the Question: The Dual-Path Model of Incongruity Resolution and Absurd Verbal Humor by fMRI

**DOI:** 10.3389/fpsyg.2017.00498

**Published:** 2017-04-24

**Authors:** Ru H. Dai, Hsueh-Chih Chen, Yu C. Chan, Ching-Lin Wu, Ping Li, Shu L. Cho, Jon-Fan Hu

**Affiliations:** ^1^National Taiwan Normal UniversityTaipei, Taiwan; ^2^National Tsing Hua UniversityHsin Chu, Taiwan; ^3^Department of Psychology, Penn State University, PennsylvaniaPA, USA; ^4^Fu Jen Catholic UniversityTaipei, Taiwan; ^5^Department of Psychology, National Cheng Kung UniversityTainan, Taiwan

**Keywords:** incongruity resolution humor, absurd humor, perspective taking, pragmatics, dual-path model

## Abstract

It is well accepted that the humor comprehension processing involves incongruity detection and resolution and then induces a feeling of amusement. However, this three-stage model of humor processing does not apply to absurd humor (so-called nonsense humor). Absurd humor contains an unresolvable incongruity but can still induce a feeling of mirth. In this study, we used functional magnetic resonance imaging (fMRI) to identify the neural mechanisms of absurd humor. Specifically, we aimed to investigate the neural substrates associated with the complete resolution of incongruity resolution humor and partial resolution of absurd humor. Based on the fMRI data, we propose a dual-path model of incongruity resolution and absurd verbal humor. According to this model, the detection and resolution for the incongruity of incongruity resolution humor activate brain regions involved in the temporo-parietal lobe (TPJ) implicated in the integration of multiple information and precuneus, likely to be involved in the ability of perspective taking. The appreciation of incongruity resolution humor activates regions the posterior cingulate cortex (PCC), implicated in autobiographic or event memory retrieval, and parahippocampal gyrus (PHG), implying the funny feeling. By contrast, the partial resolution of absurd humor elicits greater activation in the fusiform gyrus which have been implicated in word processing, inferior frontal gyrus (IFG) for the process of incongruity resolution and superior temporal gyrus (STG) for the pragmatic awareness.

## Introduction

One of the perspectives of humor processing is to perceive the incongruity in the material, resolve it and elaborate the characteristics of the protagonist to find the humor. Originating from incongruity resolution theory and comprehension-elaboration theory, this perspective has been verified by functional magnetic resonance imaging (fMRI) studies (Chan et al., 2012, 2013; Shibata et al., 2014). Among these studies, Chan et al. (2013) and Chen et al. (2017) proposed the *Three-stage Model of Humor Processing* based on the course of humor processing in incongruity resolution theory. Chan et al. (2012, 2013) used five stimuli, including incongruity-resolution jokes, unfunny, garden-path, nonsense, and nonsensical sentences, and identified three stages of humor processing by comparing the imaging data of two of them. In this model, the individual would detect the incongruity part of joke at first supported by the neural activity in the middle temporal gyrus (MTG) and middle frontal gyrus (MFG), and then reestablish and reinterpret the setup and punch line to resolve the incongruity, supported by the neural activity in the superior frontal gyrus (SFG) and inferior parietal lobe (IPL). Finally, the individual would perceive the feeling of pleasure if he/she comprehends the incongruity, supported in the neural activity in the ventromedial prefrontal cortex (vmPFC), the bilateral amygdala and the bilateral parahippocampal gyri (PHG). The three-stage humor model focuses mainly on incongruity resolution humor. The incongruity is caused by the contrast between the expectation and setup, but the content of incongruity could take place in real life, therefore, the humor can be completely resolved (Samson et al., 2009). Then, one can elaborate the characteristics and features of the protagonist from the context, and then elicit the feeling of amusement. Due to limitations of experimental design, this model only describes the processing of incongruity resolution humor in detail, while, in fact, people encounter many other types of humor in real life. To improve the generalizability of this model, we further distinguished the neural mechanisms during processing of different types of humor by using the three-stage neural circuit model as a basis.

In contrast to incongruity resolution humor, which can be fully resolved, nonsense humor can only be partially resolved because it involves an incongruity that is opposed to reality or logic. These two types of humor are categorized by the resolvability of the incongruity (Samson et al., 2009). Samson et al. (2009) suggest the incongruity involved in incongruity resolution humor is playfully resolvable and can be fully or completely resolved based on the context of the setup and the punch line; in contrast, nonsense humor can only be partially resolved and even induces new absurdities. In addition to the extent (quantity) of the resolvability, the incongruity resolution, amusement and other aspects also vary because of the nature of the incongruity. Previous research has given little attention to the processing of the two types of humor based on the three stages of humor detection, resolution and elaboration. Hence, this study explored the processing of nonsense humor based on the three-stage model of humor and compares to the processing of incongruity resolution humor. Additionally, to avoid confusing nonsense humor and nonsensical sentences (nonsensical sentences are unfunny and are semantically, grammatically and syntactically illogical) and instead of using nonsense humor, this study used absurd humor (Weller et al., 1976; Mundorf et al., 1988) to highlight the unresolvable part of humor because it involves an absurdity that cannot be completely resolved by its readers.

To date, only Samson et al. (2009) have examined the disparities between the neural mechanisms activated by incongruity resolution cartoon and those activated by absurd cartoon. In their research, stimuli were presented one by one during fMRI scanning, and participants were asked to rate the cartoons according to their comprehensibility and funniness. The results indicated that processing incongruity resolution cartoons activated brain regions in the temporo-parietal junction (TPJ), implying that it involved the process of the integration of multi-sensory information, coherence building, and inferring knowledge and anterior medial prefrontal cortex, indicating that it play a role in associating references to reality or to one’s own experiences. Clearly, different neural mechanisms were activated for processing incongruity resolution humor and absurd humor. A larger number of brain areas were activated, and the activation patterns were stronger for processing incongruity resolution humor than for absurd humor. This difference can be attributed to the incongruities in incongruity resolution humor because they elicit many assumptions from the context of the setup and punch lines, allowing one to choose the more humorous interpretation. This type of processing requires considerable mental manipulation of information (working memory), mental organization, and integration of information (Samson et al., 2009). Because of the differences in the neural substrates between pictorial and verbal humorous material (Watson et al., 2007), we applied these findings in the current study to verbal humorous materials, which are believed to have a large impact on social interaction and communication (Graham, 1995; Wanzer et al., 1996; Nezlek, 2001). Three types of ratings (surprise, comprehensibility, and funniness) were developed to reflect the three stages of humor processing.

The purpose of the current study is to construct a dual-path model of incongruity resolution and absurd humor. This model analyzes the neural mechanisms underlying the processing of these two kinds of humors based on the three-stage neural circuits in humor processing.

### Incongruity Resolution Humor

For incongruity resolution humor, incongruities are detected when a punch line deviates from one’s expectation or script formed from the content in a setup. The expectation or script can be viewed as one’s internal schema. In other words, incongruities come from the gap or distance between the punch line and the expectation or script (Hempelmann and Ruch, 2005). To fill the gap or distance between the expectation and schema, one must resolve the humor by switching to the perspective of the joke’s main protagonist. By breaking the original framework built for the expectation or script, changing to a different perspective to reinterpret the relationship between the setup and punch line becomes necessary to completely resolve the incongruities. In the affective stage of humor elaboration, Coulson and Kutas (2001) argued that the individual could connect successfully the meaning between the setup and punch line by twisting the meaning of the setup. Moreover, we provided more specific explanations that one can elaborate on the main protagonist’s personality traits or characteristics based on the storyline of the joke. In this manner, one may feel superior and experience a boom in self-esteem by derogating the main protagonist (Martin, 2007; Ferguson, 2008). Or, the tabooed content in humor could also help release one’s stress, which in turn evokes a feeling of amusement.

The following presents an example: ‘Teacher: Why are you late? Student: There was a man who lost a 100-dollar bill. Teacher: I see. Were you helping him look for it? Student: No. I was standing on it.’ When a reader reads the setup, which is the teacher’s question, ‘Were you helping him look for it?,’ he will form an expectation that the student was late for class because he was helping the man looking for the bill. However, when the reader reads the punch line, or the student’s answer, which is ‘No. I was standing on it,’ the discrepancy between the humorous content and formed expectation emerges, leading to an incongruity. To resolve such incongruity, one must read back and forth over the setup and punch line to integrate multiple pieces of information. One must also change the student’s perspective from a warm-hearted and helpful student to a greedy student to make sense of the punch line. Furthermore, to elicit stronger feeling of funny, the reader could elaborate on the student’s personality traits (he does not comply with moral ethics) to gain a sense of superiority by derogating the student (“If I were him, I would not have done the same.”). For example, one may even refer to his own experience, such as “I once spotted money on the street, but I returned it to the owner instead of keeping it.” This would then induce a feeling of enjoyment.

Previous studies have shown that resolving incongruities in incongruity resolution humor activates the TPJ, implicated in making sense of or making the coherence between the setup and punch line (Humphries et al., 2006; Samson et al., 2009; Chan et al., 2013); the precuneus, implying the area is associated with perspective taking (Ruby and Decety, 2001; D’Argembeau et al., 2007; Watson et al., 2007; Mano et al., 2009); and the areas that retrieve one’s episodic memories for comparison, such as the posterior cingulate cortex (PCC) which plays a role in retrieving episodic memories for comparison (Maddock, 1999; Alden et al., 2000; Maddock et al., 2001). Other areas activated the PHG, likely to be involved in inducing a feeling of superior and amusement (Chan et al., 2012, 2013).

### Absurd Humor

In absurd humor, the setup-induced expectation or script is inconsistent with the punch line, and the content of the punch line is simply absurd or against nature and can never occur in real life (Hempelmann and Ruch, 2005; Samson et al., 2009). To resolve such incongruity, because the punch line only provides little information for resolution (Ruch et al., 1990; Ruch and Hehl, 2007), one can only partially resolve it by processing the linguistic (phonological and semantic) aspect of the punch line. However, the illogical incongruous part remains unresolvable. It comprises the residual incongruity and serves as the absurdity element in absurd humor (Hempelmann and Ruch, 2005; Beermann and Ruch, 2009). Despite the unresolvable nature of the absurdity element, one can still be released from the residual incongruity and give the appearance of comprehending the incongruity (Pien and Rothbart, 1977; Ruch, 2001) to make the incongruity spuriously appropriate (Hempelmann and Attardo, 2011). One can then enjoy the inconsistency among the absurdity, sense and nonsense (Ruch and Hehl, 2007). The process of comprehending absurd humor with pragmatic awareness is called pseudo resolution. Because incongruities in absurd humor can only be partially resolved and most of the absurdity element stays unresolvable, pseudo resolution is also called partial resolution (Ruch, 1992; Ruch and Hehl, 2007). In the affective stage of humor elaboration, one would fail to elaborate on the main protagonist’s personality traits or characteristics from the storyline. Instead, only elaboration that is unreasonable or does not correspond to personal traits can be generated. As a result, the elicited feeling of amusement would be weaker.

For example, consider the following joke: ‘A margarine thrown out of a window by an old woman suddenly becomes a butterfly.’ When an individual reads the setup ‘A margarine (which was) thrown out of a window by an old woman,’ the individual would form an expectation that the old woman does not like the margarine or the margarine has gone bad and become inedible, so she throws it away. However, the punch line “it becomes a butterfly” deviates from that expectation and thus produces incongruity. To resolve such incongruity, the reader must process the joke on a semantic or phonological level. In this joke, processing on the semantic level resolves the incongruity as the margarine is tossed away, which is an action resembling flying. As a result, the partial incongruity is resolved. However, a margarine tossed out of a window could never turn into a butterfly. These two unrelated items cause some incongruity that cannot be resolved, that is pseudo-resolution. Furthermore, as one fails to elaborate on the personality trait of the main protagonist-the margarine-the induced feeling of amusement is weaker, or one may even find the joke confusing or unfunny. Past studies have suggested that absurd humor more often activates the lingual gyrus, fusiform gyrus, and inferior frontal gyrus (IFG) which two regions are related to word (phonological and semantic) processing and the superior temporal gyrus (STG) which region involved pragmatic awareness (Kuperberg et al., 2000; Eviatar and Just, 2006).

In sum, comprehension of the two types of humor occurs in three stages: incongruity detection, incongruity resolution, and elaboration. Despite the conclusion from Samson et al. (2009), which states that incongruity resolution and absurd humor differ only in the incongruity resolution stage, the dual-path model (**Table 1**) reveals differences in all three stages.

**Table 1:** Dual-path model of incongruity resolution and absurd humor.

	Similarity^a^	Difference	
		Incongruity-resolution humor	Absurd humor
Incongruity detection	Both detect incongruities in jokes (the left MFG and MTG)		
Resolution	Both require linguistic processing	(1) The individual would semantically process the humorous material (the right angular gyrus), take a different perspective (the precuneus), and integrate multi-sensory information in the setup and punch line (the TPJ and IPL) to form an adequate script for reinterpretation.	(1) The individual would phonologically and semantically process the humorous material (the lingual gyrus, the fusiform gyrus and the IFG); however, the absurdity element cannot be resolved, making it a partial resolution process (the IFG).
		(2) Most or all incongruities can be resolved, making it a complete resolution process.	(2) The individual can only use pragmatic awareness to meta-comprehend and interpret absurd humor (the STG), making it a pseudo-resolution process.
Humor elaboration	Both elicit a feeling of amusement, but the intensity of such feeling varies.	One retrieves event or episodic memory (the PCC) to elaborate on the personality traits of the main protagonists. By comparing the protagonists with one’s self, a feeling of superiority emerges, which in turn induces a feeling of funniness and amusement (the PHG).	One fails to elaborate on the main protagonist’s personality traits, therefore, only a feeling of confusion and absurdity can be induced.


^a^*Similarity refers to the stages that one would go through while comprehending and appreciating both humor types; however, the underlying mechanisms differ*.

Compared to three-stage model of humor which only elaborates the process of incongruity resolution humor, the dual-path model extends to the absurd humor which are prevalent in the daily life. Based on the nature of incongruity and mechanisms of resolving the incongruity of incongruity re resolution and absurd humors are entirely different, the processing of three stages would be diverse. In the incongruity detection stage, incongruities in incongruity resolution humor occur because of the gap or distance between the punch line and the expectation or script. In contrast, incongruities in absurd humor do not only stem from the reason mentioned above; they are also unnatural, and the punch line may even induce more absurdity or incongruity (Ruch et al., 1990). In the incongruity resolution stage, incongruities are resolved through the process of perspective taking and integrating multi-sensory information, a process called complete resolution. By contrast, incongruities in absurd humor can only be resolved by considering the phonological and semantic aspects of a word, without the possibility of resolving the absurd element. Thus, this process is partial resolution. Moreover, because the absurdity element does not comply with logics, such incongruity can only be resolved with meta-comprehension by pragmatic awareness. Finally, in the humor elaboration stage, one can elaborate on the personality traits of the protagonists involved in incongruity resolution humor, and those traits are typically linked to a protagonist’s weaknesses or abnormal behaviors. However, in absurd humor, only weird, ridiculous, and non-personality-related traits can be elaborated. Based on these findings, the current study aims to construct the dual-path model with support from brain imaging data to evidence that comprehension of the two types of humor differs not only in the congruity resolution stage but also in the elaboration stages. The sub-processes in each stage, including psychological mechanisms and neural circuits, will also be further illustrated.

Three types of verbal materials were designed in this study: incongruity-resolution humor, absurd humor and neutral sentences. In addition, fMRI was applied to investigate the neural substrates of incongruity resolution and absurd humor to construct the dual-path model. Our hypotheses are as follows (**Table 1**):

(1) Regarding incongruity detection, detecting incongruities in incongruity resolution and absurd humor will activate a larger number of detection related areas, including the MTG and MFG.
(2) Regarding incongruity resolution, resolving incongruities in incongruity resolution humor includes a complete resolution process, which will lead to the activation of areas related to semantic integration or coherence building, such as the TPJ and perspective-taking areas, including the precuneus. However, resolving incongruities in absurd humor includes a partial resolution process, which will activate areas related to phonological, semantic, and word processing, including the IFG and areas associated with pragmatic awareness, such as the STG.
(3) Regarding elaboration, elaborating on the personality traits of the main protagonists in incongruity resolution humor, but not in absurd humor, reflects a complete resolution process and induces a feeling of mirth. This will activate areas associated with episodic memory retrieval, including the PCC, as well as areas with activity associated with happiness, including the PHG.

## Materials and Methods

### Participants

Twenty-seven participants, aged between 21 and 30 years (*M* = 23.26, *SD* = 2.19, 15 males), participated in this study. All participants were native Chinese speakers and right-handed (as determined by the Edinburgh Handedness Inventory; Oldfield, 1971). Written informed consent was obtained from all participants prior to the scanning session. This study was approved by the Research Ethics Committee of the National Taiwan University.

### Stimuli

The current study used incongruity-resolution humor (INS-RES), absurd humor (ABS) and neutral sentences (NEU) as the experimental stimuli to explore the brain activation patterns when processing humor. Each stimulus is divided into two parts, including setup and punch line. To make the punch lines of NEU match with the ones of humorous stimuli, we rewrote the punch line of humorous stimuli to unfunny sentences. The humorous stimuli are collected from the Chinese joke database of Cheng et al. (2013) which contains incongruity-resolution jokes, absurd jokes, aggressive jokes, sexual jokes, black jokes, disgust jokes, and political jokes and so on. According to 9-point scale rating from 1 to 9, the funniness rating of incongruity-resolution and absurd jokes are on average (*M*_INC-RES_ = 4.99, *SD*_INC-RES_ = 2.33; *M*_ABS_ = 4.44, *SD*_ABS_ = 2.47).

The definition of INS-RES are the jokes violates the script developed by the setup and result in the incongruity. The readers could resolve the incongruity through the process of language, perspective taking and the process of resolution. The setup of the example is ‘Little wolf decided to be a vegetarian and father wolf does not agree with his son at all. One day, little wolf chases after a rabbit. Just a moment when father wolf feels that his son is back to normal, he hears that little wolf yells,’ the punch line of incongruity-resolution jokes is ‘Got you! Give me that carrot.’ and NEU is ‘Got you! You looks so yummy.’

The definition of ABS is the humor violates the scripts of setup and results in the incongruity. The readers could resolve the incongruity by language process and leave residual incongruity that could not resolve. The setup of ABS is ‘One day, Banana decided to go to work by bicycle. On his way home, it suddenly started to rain. When he was just about to arrive home on the mountain, he slipped and rolled all the way down the mountain. Fortunately, a man saw him and asked,’ the punch line of ABS is ‘Eggplant! Are you alright?’ and NEU is ‘Banana! Are you alright?’.

Furthermore, the current study adapted Chinese Readability Index Explorer (CRIE) to examine whether the language characteristics of stimuli are different or not. Readability refers to the level of text is comprehended by the reader. CRIE contains 31 index to assess the readability of the text at four levels including the word, semantic, syntax, and cohesion levels and with high validation (Sung et al., 2014, 2015).

A one-way ANOVA performed on the linguistic features for three types of stimuli were not significantly different. It means that the linguistic characteristics of the stimuli show no difference, therefore, we can refer the difference of brain activation patterns result from the humor mechanism instead of the linguistic characteristics. See Appendix 1 for detailed analysis on means, standard deviations and significance levels of INC-RES, ABS, and NEU on CRIE linguistic features.

After a strict filtering process, 12 texts for each stimulus type; thus, a total of 48 texts were selected for the experiment. The setup of each type of stimuli contained 39–42 words (*M*_INC-RES_ = 39.81, *SD*_INC-RES_ = 3.62; *M*_ABS_ = 41.33, *SD*_ABS_ = 2.93; *M*_NEU_ = 39.94, *SD*_NEU_ = 3.57), and the punch lines contained 10–11 words (*M*_INC-RES_ = 10.36, *SD*_INC-RES_ = 1.73; *M*_ABS_ = 10.67, *SD*_ABS_ = 2.42; *M*_NEU_ = 10.26, *SD*_NEU_ = 1.75).

### Experiment Paradigm

**FIGURE 1:**
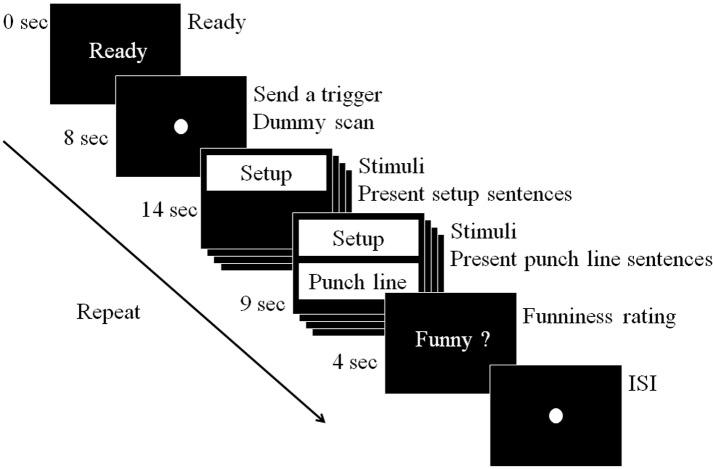
**Functional magnetic resonance imaging (fMRI) scanning procedure**.

Event-related fMRI was used as the experimental paradigm in this study. The experiment was a within-subject design. At the beginning of the session, a screen displayed *Ready*, and the subjects were instructed to stare at a dot that was presented on the screen for 8 s. Then, 48 sets of word stimuli (INC-RES, ABS, and NEU) were presented randomly on the screen such that subjects did not develop a tendency to answer the questions. A setup stimulus then appeared on the screen for 14 s, followed by a punch line stimulus that appeared immediately below the setup for 9 s. The setup remained on the screen, allowing the participants to look for clues in the context to resolve incongruities. Afterward, a funniness rating of 1–4 was shown on the screen for 4 s, followed by a jittered inter-stimulus interval (ISI) of 3.1 to 9.9 s (*M* = 6.2) to allow for emotional recovery. During ISIs, participants were asked to fixate their vision on a dot presented on the screen, without closing their eyes, until the run was completed. Overall, participants could take three 2-min closed-eye breaks between the four runs until the experiment ended (**Figure 1**). Each run was 6 min and 8 s long. Including a total of four runs and three breaks, the total experiment duration was 33 min and 1 s, or approximately 33 min.

After showing the setup and punch line of each condition in the scanning session, a four-point Likert scale was used to evaluate a subject’s perceptions. To obtain a more specific rating response, the subjects were asked to evaluate the material after a scanning session. The same materials were presented in the same order as that presented in the scanning session. Participants were asked to recall their reading experience during the formal experiment and rate each stimulus from 1 to 9, with 9 being the highest score, in terms of surprise, comprehensibility, and funniness (The correlation of funniness rating between the scanning session and post-scanning session is significantly positive (*r*_In-scan_ _surprise_ · _Post-scan_ _surprise_ = 0.87, *r*_In-scan_ _comprehensibility_ · _Post-scan_ _comprehensibility_ = 0.88, *r*_In-scan_ _funniness_ · _Post-scan_ _funniness_ = 0.82). Therefore, the current study provided the results of post scanning in Section “Behavioral Results.”

### Neuroimaging Data Acquisition

The experiment was conducted using a 3T (3 Tesla) Siemens Megnetom Skyra fMRI scanner with a quadrature transmit-receive-head coil for 32 channels. The experiment program was written with E-prime software and was coupled with the vendor’s handheld fiber optic response device for fMRI, which included four buttons. The visual stimuli were projected from a computer screen onto a mirror positioned in front of the participant. The T1 anatomical imaging was performed prior to the formal experiment and included a high-resolution T1-weighted structural image, which was used to match the activated and connected brain regions [pulse sequence, 3D-MPRAGE; repetition time (TR) = 1,560 ms; echo time (TE) = 3.68 ms; flip angle (FA) = 15°; matrix size = 256 × 256; field of view (FOV) = 256 mm × 256 mm; slice number = 192; thickness = 1 mm; spatial resolution = 1 mm × 1 mm × 1 mm; scanning time = 3.5 min]. For T2 coronal view scanning, the following parameters were used: pulse sequence, MPRAGE = 256; TR = 5,920 ms; TE = 102 ms; FA = 90°; matrix size = 256 × 256; FOV = 256 mm × 256 mm; slice number = 34; thickness = 3 mm. Functional imaging included a T2-weighted gradient and EPI pulse sequence in each run to magnify the contrast of blood-oxygen-level dependent (BOLD) signals (EPI: TR = 2,000 ms; TE = 24 ms; FA = 90°; matrix size = 64 × 64; FOV = 256 mm × 256 mm; 34 axial views in 1 TR, thickness = 3 mm; voxel size = 4 mm × 4 mm × 3 mm; no gap). The anterior commissure-posterior commissure line (AC-PC line) was targeted during scanning to capture the image of whole brain.

### Data Analysis

The data collected included behavioral and neuroimaging data. For behavioral data, a one-way repeated-measures ANOVA were used to analyze the differences between incongruity resolution, absurd humor and neutral sentences in terms of surprise, comprehensibility, and funniness were rated after scanning.

The imaging data analysis was divided into pre-processing and statistical analysis. SPM8 (Statistical Parametric Mapping; Wellcome Department of Cognitive Neurology, London, UK) software was used to process and analyze fMRI images. The pre-processing analysis consisted of slice timing, realignment, coregistration, normalization, and smoothing. Regarding slice timing, most pulse sequences collect data by interleaved slicing (odd-numbered slices first, followed by even-numbered slices) to reduce cross-slice activation. Hence, the interpolation method was used to correct the interleaved slice timing and minimize temporal errors. The first slice was used as the reference slice for alignment. With respect to realignment, each participant’s head was realigned to prevent analysis of artifacts. If a subject’s head motion in each run was larger than one voxel, or approximately 3 mm, for any of the three translation parameters (x-, y-, z-axes) or three rotation parameters (x-y, y-z, and x-z planes), the subject’s data were removed the analysis. For co-registration, T1- and T2-images and EPI were sequentially aligned and calibrated, with T1- and T2-images chosen as source images. In terms of normalization, mathematical expansion, compression, and deformation were employed to match each brain image to the standardized EPI template created using Montreal Neurological Institute (MNI) coordinates. In one segment in the pre-processing analysis, a subject’s gray and white matter images could be separated for alignment during normalization. Finally, smoothing was employed to increase the signal-to-noise ratio (SNR). A Gaussian kernel filter with an 8 mm full-width at half-maximum (FWHM) was applied in this study. The statistical analysis focused on changes in signals to determine the temporal relationships between stimuli and brain region activation. The dynamics of activation was investigated by setting a threshold value to distinguish between the activated and inactivated areas. The activated regions were then revealed in detail with high-resolution anatomical imaging.

For single-subject analyses, the different event types (INS-RES, ABS, and NEU) were defined. These onset functions were then convolved with the canonical hemodynamic response function (HRF) and its temporal derivative to capture the variability in the latencies of BOLD responses. Group data were smoothed, and the individual subject contrast images were entered into a whole-brain, random-effects analysis to determine whether there was significant activation by a contrast. The analysis of the parametric modulation was analyzed using paired-sample *t*-test. Group results for each of the two main effect contrasts (INS- REC contrast NEU, ABS contrast NEU, INS- REC contrast ABS and ABS contrast INS- REC) are reported. In order to avoid the non-independence error of region of interest (ROI) analysis, the current study adopted the split-half analysis. We used the odd runs data to identify candidate regions of humor processing, and the even runs data to estimate the BOLD signal change (Kriegeskorte et al., 2009; Vul and Kanwisher, 2010). Since that we got the candidate regions of humor processing by the half data, small volume correction (SVC) were conducted at extent threshold of *p* < 0.001 with a sphere of 8 mm radius.

## Results

### Behavioral Results

A one-way repeated-measures ANOVA analysis showed that participants’ surprise ratings was significantly different [*F*(2,52) = 34.12, *p* < 0.001, η^2^ **=** 0.57] and Bonferroni *post hoc* tests revealed that INC-RES and ABS were significantly more surprising than NEU. Additionally, participants’ funniness ratings was significantly different [*F*(2,52) = 85.12, *p* < 0.001, η^2^ = 0.77] and Bonferroni *post hoc* tests revealed that the most funny condition is INC-RES, then ABS and the less funny condition is NEU. Lastly, participants’ comprehensibility ratings was significantly different [*F*(2,52) = 1.88, *p* > 0.05, η^2^ = 0.07]. The results are listed in **Table 2**.

**Table 2:** One-way repeated-measures ANOVA analysis for ratings of INC-RES, ABS, and NEU.

	INC-RES Mean (*SD*)	ABS Mean (*SD*)	NEU Mean (*SD*)	*F*	Effect size	*Post hoc*
Surprise	6.18 (1.66)	5.66 (1.71)	4.46 (1.58)	34.12^∗∗∗^	0.57	INC-RES, ABS > NEU
comprehensibility	7.83 (1.09)	7.66 (1.18)	7.33 (1.23)	1.88	0.07	
Funniness	6.48 (1.56)	6.00 (1.90)	3.38 (1.53)	85.12^∗∗∗^	0.77	INC-RES > ABS > NEU


*The scale of rating is from 1 to 9, with 9 being the highest score, in terms of surprise, comprehensibility, and funniness. ^∗∗∗^*p* < 0.001*.

### fMRI Results

#### Brain Regions Activated by Incongruity Resolution Humor

After subtracting the activation for reading neutral sentences from that of reading absurd humor, incongruity resolution humor significantly activated the left precuneus extending to the right paracentral lobule and right cingulate gyrus, the right MTG extending to the right SFG, the right angular gyrus extending to the right IPL, the left PHG extending to the right culman, and the right PHG extending to the right amygdala (see **Table 3**).

**Table 3:** Voxel coordinates in MNI space and associated z scores showing BOLD activation for INC-RES (minus NEU) vs. ABS verbal humors (minus NEU) as well as the subtraction (INC-RES minus NEU)-(ABS minus NEU) and (ABS minus NEU)- (INC-RES minus NEU).

Regions	BA	Voxels	*z* score	Coordinates		
				*X*	*Y*	*Z*
**INC-RES minus NEU**
L-PreC, R-paracentral lobule, R-cingulate gyrus	7/5/31	1204	3.56	-9	-58	52
R-MFG, R-SFG, R-SFG	10/9/10	162	3.44	33	59	22
R-angular gyrus, R-IPL	39/40	430	3.35	48	-70	37
L-PHG, R-culmen	30	224	3.34	-30	-52	4
R-PHG, R- amygdala	34	83	3.30	15	-13	-17
**ABS minus NEU**
L-STG	22/39	34	2.82	-63	-55	16
L-IFG	9	13	1.96	-51	20	25
L-fusiform gyrus	18	10	1.88	-24	-91	-14
**(INC-RES minus NEU) – (ABS minus NEU)**
L-PCC, L-PreC, R-paracentral lobule	23/31/31	941	3.55	-9	-37	25
R-IPL, R- TPJ	40/39	408	3.54	60	-37	49
L-SFG, L-MFG	10/11	25	3.32	-18	53	-8
R-PHG	28	60	3.22	15	-16	-14
R-precentral gyrus	6	17	3.00	63	5	28
**(ABS minus NEU) – (INC-RES minus NEU)**
L-fusiform gyrus	18	209	3.85	-24	-91	-14
R-lingual gyrus	18	262	3.80	24	-91	-8
L-IFG, L-precentral gyrus, L-MFG	45/6/9	293	3.38	-54	23	4
L-STG, L-STG, L-MTG	22/39/22	111	2.86	-63	-52	10
L-fusiform gyrus	37	21	2.82	-39	-58	-14


*Statistical threshold for illustrative purposes was chosen as *p* < 0.001 (uncorrected at the peak) or extent (cluster level) threshold of *p* < 0.05 (FWE corrected) at the voxel level with a cluster size greater than or equal to 10 voxels. INC-RES, incongruity resolution verbal humors; ABS, absurd verbal humors; NEU, unfunny general sentences; PreC, precuneus; MFG, middle frontal gyrus; SFG, superior frontal gyrus; IPL, inferior partial lobe; TPJ, temporo-parietal junction; PHG, parahippocampal gyrus; STG, superior temporal gyrus; IFG, inferior frontal gyrus; PCC, posterior cingulate gyrus; MTG, middle temporal gyrus*.

#### Brain Region Activated by Absurd Humor

When subtracting the activation for reading neutral sentences from that for reading absurd humor, absurd humor significantly activated the left STG, left IFG, and left fusiform gyrus (see **Table 3**).

#### Brain Region Activated by Incongruity Resolution Humor vs. Absurd Humor

**FIGURE 2:**
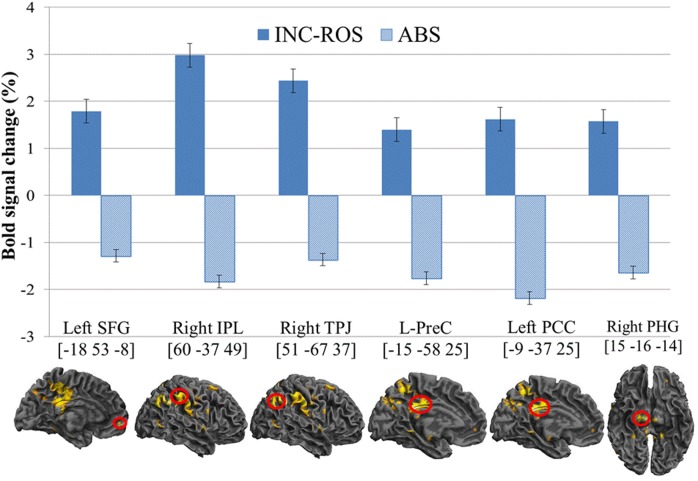
**Enhanced BOLD signal activation for incongruity resolution contrast absurd verbal humor.** Incongruity resolution verbal humor demonstrated stronger activation in the left MFG (BA 11), left SFG (BA 10), right IPL (BA 40), right TPJ (BA 39), left PreC (BA 31), left PCC (BA 23), and right PHG (BA 28). MFG, middle frontal gyrus; SFG, superior frontal gyrus; IPL, inferior partial lobe; TPJ, temporo-parietal junction; PreC, precuneus; PCC, posterior cingulate gyrus; PHG, parahippocampal gyrus; INC-RES, incongruity resolution humor; ABS, absurd humor.

When subtracting the activation for reading absurd humor from that for reading incongruity resolution humor, the left PCC extending to the left precuneus and left paracentral lobule, the right IPL extending to the right angular gyrus (TPJ), and the right SFG extending to the left MFG, right PHG and right precentral gyrus were significantly activated (see **Table 3** and **Figure 2**).

#### Brain Region Activated by Absurd Humor vs. Incongruity Resolution Humor

**FIGURE 3:**
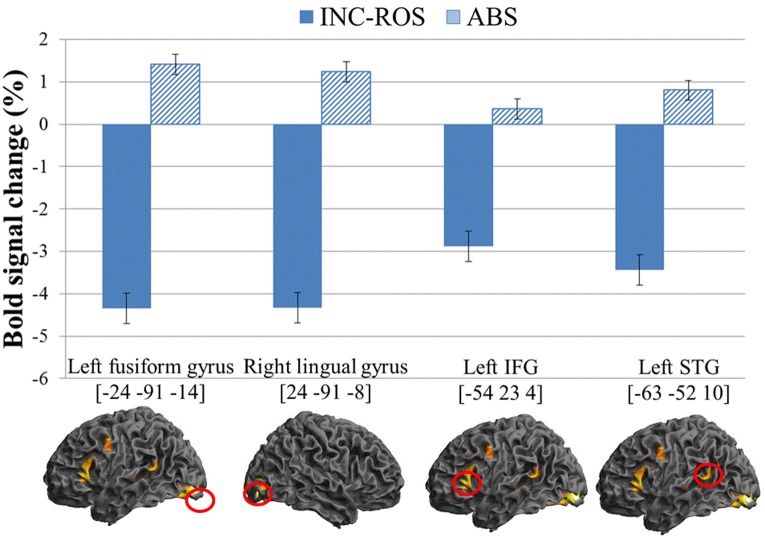
**Enhanced BOLD signal activation for absurd contrast incongruity resolution verbal humor.** Absurd verbal humor demonstrated enhanced activation in the left fusiform gyrus (BA 18), right lingual gyrus (BA 18), left IFG (BA 45), and left STG (BA 22). IFG, inferior frontal gyrus; STG, superior temporal gyrus; INC-RES, incongruity resolution humor; ABS, absurd humor.

After subtracting the activation for reading incongruity resolution humor from that for reading absurd humor, the left fusiform gyrus, right lingual gyrus, left IFG extending to the left precentral gyrus and left MFG, and left STG extending to the left MTG were significantly activated (see **Table 3** and **Figure 3**).

## Discussion

This study used fMRI to examine the neural mechanisms underlying the processing of incongruity resolution and absurd verbal humor and to establish the dual-path model for the two humor types. Our model and imaging data imply that in addition to the differences observed in the incongruity resolution stage reported in previous studies, the mechanisms and neural circuits vary for the other two stages (incongruity detection and elaboration). In the following paragraphs, the behavioral results are discussed first, followed by the brain imaging results and the dual-path model of incongruity resolution and absurd verbal humor based on the integrated data.

### Behavioral Results

According to the findings from our behavioral data, individuals found incongruity resolution verbal humor considerably more surprising than absurd verbal humor. A study (Alden et al., 2000) on humorous TV commercials indicated that when a viewer is highly familiar with the scenario in a commercial, a high degree of incongruity induces surprising feelings. In other words, how familiar one’s schema is with the commercial will affect the level of surprise induced by the incongruity. The schema is the viewer’s expectation or perspective shaped by the viewer’s experience and knowledge, which is highly related to oneself. For incongruities in incongruity resolution verbal humor, in which the punch line notably deviates from or is against one’s schema, the induced feeling of surprise should be strong (for example, a warm-hearted and helpful student vs. a greedy student). By comparison, incongruities in absurd verbal humor are illogical (for example, the margarine thrown out of the window becomes a butterfly). As natural rules are less akin to oneself than the schema or expectation, the incongruities in absurd verbal humor actually elicit weaker feelings of surprise than incongruity resolution humor.

In addition to the surprise rating, participants found incongruity resolution verbal humor funnier than absurd humor, either during or after the scanning session. Processing humor requires two components: a feeling of surprise and consistency (Bartolo et al., 2006; Forabosco, 2008). The surprise feeling is due to the inconsistency between one’s expectation or script formed from the setup and the punch line. Our behavioral data demonstrated that incongruity resolution verbal humor was significantly more surprising than absurd verbal humor, and the incongruities in the former could be completely resolved. Hence, incongruity resolution verbal humor was funnier. Conversely, absurd verbal humor was less surprising with only partially resolvable incongruities and was thus not as funny.

### Imaging Results

With regard to the three stages of humor processing, despite their differences in structure and neural mechanisms, processing incongruity resolution and absurd humor both activate the same brain region, namely, the left middle frontal gyrus (L-MFG), an area responsible for the short-term storage and processing of working memory (Leung et al., 2002), as well as the phonological and semantic processing of Chinese words (Liu et al., 2006). Thus, despite their different structures (or resolvability levels), one needs to use working memory to store messages in the short term for both humor types to make comparisons with information in long-term memory. Moreover, as the two types of humor are both verbal, one must also process the content on phonological and semantic levels.

#### Neural Correlates of Incongruity Resolution Verbal Humor

As noted above, we hypothesized that the different structures of incongruity resolution and absurd verbal humor would activate different brain regions. Our findings indicated that incongruity resolution verbal humor showed greater activation in the left PCC extending to the left precuneus and left paracentral lobule, the right IPL extending to right TPJ, the right SFG extending to left MFG, right PHG, and right precentral gyrus than absurd verbal humor.

While processing incongruity resolution humor, one forms an expectation or script based on the setup. However, incongruities emerge when one finds that the punch line is inconsistent with the expectation or script. To resolve such incongruities, one must establish a proper script that fits the punch line. Our results demonstrated that certain brain regions in the right hemisphere were activated by processing incongruity resolution humor, including the right IPL. Previous brain imaging studies have shown that the IPL is linked to semantic comprehension and integration (Humphries et al., 2006), causality building between the setup and punch line (Samson et al., 2009), and incongruity resolution (Chan et al., 2013). Therefore, it is perceivable that to resolve incongruities, one must encode the words and process their semantic aspects and concepts in verbal humor and look for causal relations between the setup and punch line to form an appropriate script. Meanwhile, the right IPL pertain to the TPJ. The TPJ is related to the integration of multiple types of information, coherence building (Ferstl and von Cramon, 2002; Samson et al., 2009), and incongruity resolution of humor (Samson et al., 2009; Vrticka et al., 2013). Therefore, the TPJ may be activated by incongruity resolution verbal humor because one must build the consistency between the setup and punch line to form an appropriate script for reinterpretation. As incongruities in absurd verbal humor cannot be resolved or can only be partially resolved (Ruch, 1992), one is neither able to effectively integrate multiple information in the verbal humor nor able to build the consistency between the setup and punch line. Thus, the TPJ is not activated more strongly during absurd verbal humor comprehension.

Our results showed that the precuneus is more strongly activated in incongruity resolution verbal humor than in absurd verbal humor. The left precuneus is related to the ability to assume different perspectives (Ruby and Decety, 2001; D’Argembeau et al., 2007; Watson et al., 2007; Mano et al., 2009) and semantic processing (Cabeza and Nyberg, 2000). We suggest that to build consistency between the setup-induced expectation or script and the punch line during the incongruity resolution stage, one must switch to the protagonist’s perspective to form an appropriate script. As a result, the punch line would match with the setup (Forabosco, 2008), and the incongruity is resolved. In other words, the ability to assume perspectives is more necessary to resolve incongruities in incongruity resolution verbal humor than in absurd verbal humor, as a protagonist in absurd verbal humor is rather common and ordinary, such as the margarine. Because one cannot take a different perspective for an ordinary protagonist, the precuneus is not activated more strongly by processing absurd verbal humor.

Incongruity resolution verbal humor also significantly activates the left PCC and PHG. Activation of the PCC is related to the coexistence of several psychological conditions (Forabosco, 2008) and activation of the PHG is associated with the affective element in humor processing (Chan et al., 2012, 2013). When one changes between different perspectives to find an appropriate script to comprehend incongruity resolution verbal humor, he or she experiences the coexistence of several psychological conditions. As a result, the PCC is activated more strongly in incongruity resolution verbal humor. Empirical studies on the PCC have mainly been performed with patients with Alzheimer’s disease, and these studies indicate the PCC is more closely linked to learning and memory (Maddock et al., 2001; Minoshima et al., 2004; Zhou et al., 2008). Other research has shown that the PCC is related to event memory (Maddock, 1999) and episodic memory (Andreasen et al., 1995; Maddock et al., 2001). Maddock et al. (2001) used familiar names as cues for retrieving memories to study episodic memory in healthy individuals and found that the memories retrieved included both semantic and event memories. Therefore, the PCC is activated while comprehending incongruity resolution verbal humor because the viewer retrieved long-term memories that were related to the protagonist or events in the joke. According to superiority theory (Martin, 2007; Ferguson, 2008), when one compares his retrieved memories with the protagonists or events in a joke and finds the protagonists foolish or funny, one would feel a boom in dignity and superiority. The right PHG is then activated, leading to a feeling of amusement.

#### Neural Correlate of Absurd Verbal Humor

In contrast to incongruity resolution verbal humor, absurd verbal humor showed increased activation in the left fusiform gyrus, right lingual gyrus, left IFG extending the left precentral gyrus and left MFG, and the left STG extending to the left MTG compared with incongruity resolution verbal humor.

The brain imaging data showed that areas activated by absurd humor were mainly located in the left hemisphere. Activation of different regions between the two hemispheres indicates different humor comprehension processes (Bihrle et al., 1986; Marinkovic et al., 2011). Left brain activation is mainly connected with detecting incongruities in humorous stimuli. As noted above, unlike incongruities in incongruity resolution verbal humor, which are against one’s expectation or schema, incongruities in absurd verbal humor are against nature and more ridiculous and eccentric (Ruch, 1992). Thus, based on our results, one detected more and stronger incongruities while reading absurd verbal humor; thus, greater activation was observed in the left hemisphere. When such incongruities were detected, the reader used more cognitive resources to find clues in the text, though limited, that could be used for interpretation.

When incongruities are detected in absurd verbal humor, one must resolve the incongruities to attain comprehension. According to brain imaging studies, STG and IFG, which show greater activation during the comprehension of absurd verbal humor, are considered to be related to incongruity detection (Moran et al., 2004; Bartolo et al., 2006; Bekinschtein et al., 2011). IFG is related to typical phonological and semantic processing (Shaywitz et al., 1995; Goel and Dolan, 2001; Heim et al., 2003; Nixon et al., 2004; Costafreda et al., 2006; Meinzer et al., 2009; Peramunage et al., 2011), adjusting linguistic comprehension, decoding stimuli (Azim et al., 2005), and processing humor and semantic ambiguity (Bekinschtein et al., 2011). In addition, the fusiform gyrus and lingual gyrus are also activated more strongly during absurd humor processing. The fusiform gyrus, which is also known as the visual word form area (VWFA), involves the processing of characters or words (McCandliss et al., 2003; Dehaene and Cohen, 2011). One study used Positron emission tomography (PET) to examine the effect of word length and visual contrast on brain area activation during reading and found that the fusiform gyrus and lingual gyrus were both involved in word processing; however, their mechanisms differed, with the former processing specific features in characters and the latter processing the overall shape of characters (Mechelli et al., 2000). Activation of the STG is also related to linguistic comprehension (Hécaen and Albert, 1979) or, more specifically, to pragmatic awareness (Kuperberg et al., 2000; Eviatar and Just, 2006). Based on the findings in previous research, we reason that because incongruities in absurd verbal humor are illogical and one is unable to form an appropriate script by changing perspectives, one can only partially resolve the incongruities by processing at the word, phonological, and semantic levels. The remaining unresolvable incongruities require pragmatic awareness. In short, the pragmatic function of humor is to provoke enjoyable feelings through funny and stimulating expression to create positive social interaction. Hence, in contrast to incongruity resolution verbal humor, absurd verbal humor activates brain regions that are mainly involved in word, phonological, and semantic processing, as well as pragmatic awareness during the incongruity resolution stage.

## Conclusion

The three stages of processing incongruity resolution and absurd verbal humor recruit distinct neural circuits. Previous research (Samson et al., 2009) stated that the difference between the two humor types lies in the quantity of resolvable incongruity; thus, the neural difference only occurs in the incongruity resolution stage. In terms of the quality of resolvable incongruity, Bartolo et al. (2006) compared the brain imaging data for humorous and non-humorous cartoons and found that incongruity resolution activated the right IFG, left STG, left MTG and left cerebellum, which, as suggested by previous research, are related to intention attribution. Thus, they claimed that the process of incongruity resolution is the process of intention attribution. However, this conclusion might be too decisive, as it was drawn from the comparison of humorous and non-humorous joke stimuli. In fact, one experiences incongruity resolution while comprehending various types of humor, and their resolution strategies vary depending on the humor type. Our study indicates the recruiting distinct neural circuits for processing incongruity resolution and absurd verbal humor can be attributed to their different structural characteristics, or the resolvability of their incongruities, and different resolution approaches. Complete resolution processing for incongruity resolution humor means that an individual processes the linguistic (phonological, semantic, and conceptual) aspects of humorous material while simultaneously using the strategy of taking perspectives. Whereas partial resolution for absurd humor, as the incongruities are against nature and too absurd, only allows one to process on word, phonological, and semantic levels. The residual incongruity can only be processed with pragmatic awareness (knowing that the purpose of the text is to make one laugh). Although previous studies focused on the quantitative differences in incongruity resolvability in the two humor types, this study further illustrates the qualitative differences in complete resolution of incongruity resolution humor and partial resolution in absurd humor with neural imaging data.

**FIGURE 4:**
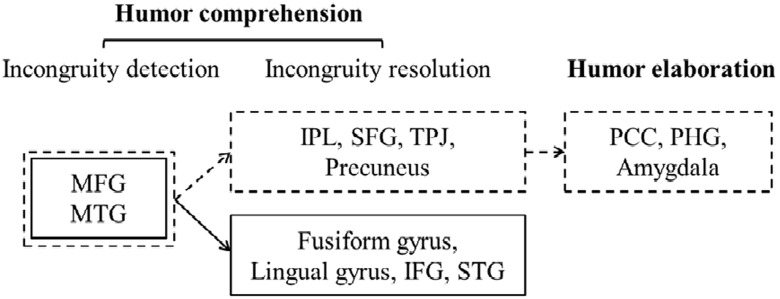
**The dual-path model of incongruity-resolution and absurd verbal humor.** The figure indicates the neural circuits of incongruity-resolution and absurd verbal humor. 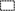 refers that the neural circuits of incongruity-resolution and absurd verbal humor and 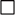 refers that the neural circuits of and absurd verbal humor.

Supported by our brain imaging data, the dual-path model of incongruity resolution and absurd verbal humor explains the difference in psychological process and neural circuits (see **Figure 4**). In the incongruity resolution stage, incongruities in incongruity resolution verbal humor can be resolved with the building the coherence and causality relationship between the setup and punch line and the ability to take perspectives. Therefore, incongruity resolution verbal humor showed increased activation in the right IPL, right TPJ and left precuneus. By contrast, incongruities in absurd verbal humor can only be partially resolved with low-level (word, phonological, and semantic) linguistic processing; the interpretation of residual incongruity is left to pragmatic awareness. Therefore, absurd verbal humor shows stronger activation in the left fusiform, lingual gyrus, IFG and STG. In the humor elaboration stage, one can elaborate on the protagonist and events described in incongruity resolution verbal humor to activate the left PCC and right PHG, involving the first is related to retrieving autobiographic or event memory and the second is involved the feeling of amusement and funniness. In conclusion, thedual-path model illustrates the sub-processes of the three stages of comprehending incongruity resolution and absurd verbal humor. It also distinguishes the underlying psychological mechanisms and neural circuits. Based on this model, future research can further investigate whether the inner mechanisms for comprehending the two humor types are affected by other variables, such as gender and autism spectrum disorder.

## Author Contributions

SC and J-FH contributed the research ideas to the study and YC and C-LW contributed the fMRI design and analysis to implement the experiment. H-CC and PL contributed to the organization of this paper. RD contributed in the analysis and writing of this study.

## Conflict of Interest Statement

The authors declare that the research was conducted in the absence of any commercial or financial relationships that could be construed as a potential conflict of interest.
