# Supplementary material for: To Resolve or Not To Resolve, that Is the Question: The Dual-Path Model of Incongruity Resolution and Absurd Verbal Humor by fMRI
Source: Front Psychol. 2017 Apr 24;8:498. doi: 10.3389/fpsyg.2017.00498 (PMC5402715; doi:10.3389/fpsyg.2017.00498)
Supplement: Supplementary file 1 [file Data_Sheet_1.DOCX]

Appendix 1. Means, standard deviation and significance levels of INC-RES, ABS and NEU on CRIE linguistic features

| CRIE feature | | Word level | | | | | | | | Syntax level | | | | |
| --- | --- | --- | --- | --- | --- | --- | --- | --- | --- | --- | --- | --- | --- | --- |
|  |  | Words | Verbs | Difficult words | Few-stroke characters | Moderate- stroke  characters | Logarithm of the content-word  frequency | Two character words | Three character words | Average sentence length | Simple sentence ratio | Modifiers per NP | Sentences with complex structure | Parallelism |
| Setup | INS-RES HUM | 39.81  (3.62) | 11.14  (2.46) | 14.44  (3.11) | 41.33  (4.60) | 19.47  (4.23) | 8.88  (0.57) | 17.33  (3.23) | 1.67  (1.67) | 8.28  (2.99) | 0.69  (0.33) | 0.26  (0.22) | 8.42  (6.55) | 0.00  (0.00) |
|  | ABS HUM | 41.33  (2.93) | 11.58  (2.61) | 16.08  (3.15) | 44.00  (7.06) | 20.75  (5.34) | 9.12  (0.76) | 17.67  (3.68) | 2.42  (1.44) | 9.44  (1.75) | 0.45  (0.38) | 0.30  (0.24) | 11.83  (6.62) | 0.00  (0.00) |
|  | NEU | 39.94  (3.57) | 11.17  (2.44) | 14.71  (2.99) | 41.40  (4.67) | 19.54  (4.29) | 8.89  (0.56) | 17.43  (3.28) | 1.63  (1.61) | 8.35(3.00) | 0.69  (0.33) | 0.26  (0.22) | 8.23  (6.39) | 0.00  (0.00) |
|  | *F* | 0.90 | 0.16 | 1.31 | 1.40 | 0.41 | 0.80 | 0.05 | 1.17 | 0.81 | 2.53 | 0.18 | 1.51 | - |
| Punch  line | INS-RES HUM | 10.36  (1.73) | 2.75  (1.30) | 3.92  (1.30) | 10.92  (2.48) | 5.08  (2.14) | 8.88  (1.06) | 4.36  (1.33) | 0.58 (0.73) | 9.54  (2.09) | 1.00  (0.00) | 0.19  (0.36) | 2.78  (3.16) | 0.00  (0.00) |
|  | ABS HUM | 10.67  (2.42) | 2.33  (1.50) | 3.83  (1.40) | 10.83  (3.04) | 4.67  (2.77) | 8.92  (0.94) | 3.83  (1.11) | 0.58  (0.79) | 9.88  (3.36) | 0.92  (0.29) | 0.31  (0.45) | 4.17  (3.76) | 0.00  (0.00) |
|  | NEU | 10.26  (1.75) | 2.77  (1.21) | 3.89  (1.60) | 10.74  (2.29) | 5.29  (1.64) | 8.99  (0.94) | 4.89  (1.53) | 0.40  (0.60) | 9.59  (2.11) | 0.97  (0.17) | 0.25  (0.43) | 2.69  (3.17) | 0.00  (0.00) |
|  | *F* | 0.22 | 0.56 | 0.02 | 0.04 | 0.41 | 0.10 | 2.89 | 0.71 | 0.10 | 1.35 | 0.46 | 1.00 | - |

Appendix 1. (continued) Means, standard deviation and significance levels of INC-RES, ABS and NEU on CRIE linguistic features

| CRIE feature | | Semantic level | | | | | Cohesion level | | | | | | | |
| --- | --- | --- | --- | --- | --- | --- | --- | --- | --- | --- | --- | --- | --- | --- |
|  |  | Content  words | Negatives | Sentences with complex semantic categories | Number of complex semantic categories | Intentional words | First personal pronouns | Third personal pronouns | Conjunctions | Positive conjunctions | Negative conjunctions | Condition conjunction | Purpose conjunctions | Figure of speech |
| Setup | INS-RES HUM | 34.08  (3.32) | 0.58  (1.16) | 3.53  (1.54) | 1.22  (0.67) | 0.14  (0.35) | 0.86  (1.07) | 0.53  (0.94) | 0.75  (0.81) | 0.39  (0.55) | 0.33  (0.59) | 0.00  (0.00) | 0.00  (0.00) | 0.00  (0.00) |
|  | ABS HUM | 33.33  (2.57) | 0.33  (0.49) | 2.67  (1.23) | 0.76  (0.36) | 0.17  (0.39) | 0.08  (0.29) | 0.67  (0.65) | 0.92  (1.08) | 0.75  (0.87) | 0.17  (0.39) | 0.08  (0.29) | 0.00  (0.00) | 0.17  (0.58) |
|  | NEU | 34.26  (3.28) | 0.63  (1.17) | 3.46  (1.50) | 1.20  (0.65) | 0.11  (0.32) | 0.80  (1.02) | 0.49  (0.95) | 0.80  (0.83) | 0.43  (0.61) | 0.34  (0.59) | 0.00  (0.00) | 0.00  (0.00) | 0.00  (0.00) |
|  | *F* | 0.37 | 0.33 | 1.61 | 2.62 | 0.11 | 3.022 | 0.18 | 0.17 | 1.56 | 0.47 | 3.11 | - | 3.11 |
| Punch  line | INS-RES HUM | 8.36  (1.93) | 0.03  (0.17) | 0.69  (0.71) | 0.16  (0.24) | 0.06  (0.23) | 0.50  (0.56) | 0.19  (0.40) | 0.28  (0.45) | 0.17  (0.38) | 0.08  (0.28) | 0.00  (0.00) | 0.00  (0.00) | 0.00  (0.00) |
|  | ABS HUM | 8.25  (2.05) | 0.08  (0.29) | 0.83  (0.58) | 0.22  (0.27) | 0.00  (0.00) | 0.17  (0.39) | 0.42  (0.51) | 0.50  (0.67) | 0.50  (0.67) | 0.00  (0.00) | 0.00  (0.00) | 0.00  (0.00) | 0.00  (0.00) |
|  | NEU | 8.57  (1.91) | 0.14  (0.36) | 0.66  (0.59) | 0.16  (0.21) | 0.00  (0.00) | 0.40  (0.60) | 0.17  (0.38) | 0.26  (0.51) | 0.17  (0.38) | 0.09  (0.37) | 0.03  (0.17) | 0.00  (0.00) | 0.00  (0.00) |
|  | *F* | 0.17 | 1.52 | 0.34 | 0.33 | 1.33 | 1.60 | 1.68 | 1.08 | 3.01 | 0.39 | 0.68 | - | - |
